# Effects of Level of Retrieval Success on Recall-Related Frontal and Medial Temporal Lobe Activations

**DOI:** 10.1155/2002/514313

**Published:** 2002-11-01

**Authors:** Daniela Montaldi, Andrew R. Mayes, Anna Barnes, Donald M. Hadley, Jim Patterson, David J. Wyper

**Affiliations:** ^1^ Department of Psychology Eleanor Rathbone Building University of Liverpool Liverpool L69 3BX UK; ^2^ Departments of Clinical Physics and Clinical Neuroradiology Institute of Neurological Sciences Southern General Hospital Glasgow Scotland UK

## Abstract

Brain dedicated single photon emission computed tomography (SPECT) was used to compare the neuroactivation produced by the cued recall of response words in a set of studied word pairs with that produced by the cued retrieval of words semantically related to unstudied stimulus words. Six of the 12 subjects scanned were extensively trained so as to have good memory of the studied pairs and the remaining six were minimally trained so as to have poor memory. When comparing episodic with semantic retrieval, the well-trained subjects showed significant left medial temporal lobe activation, which was also significantly greater than that shown by the poorly trained subjects, who failed to show significant medial temporal lobe activation. In contrast, the poorly trained subjects showed significant bilateral frontal lobe activation, which was significantly greater than that shown by the well-trained subjects who failed to show significant frontal lobe activation. The frontal activations occurred mainly in the dorsolateral region, but extended into the ventrolateral and, to a lesser extent, the frontal polar regions. It is argued that whereas the medial temporal lobe activation increased as the proportion of response words successfully recalled increased, the bilateral frontal lobe activation increased in proportion to retrieval effort, which was greater when learning had been less good.

## 1. Introduction

It is well known that lesions of the medial temporal lobes (MTL) and the prefrontal cortex (PFC) (see [30]), whether left- or right-sided or bilateral, disrupt the acquisition of memories about episodes and facts and the retrieval of memories about some episodes and less well-learned facts (see [15]). In contrast, retrieval of well-established factual memories seems to be immune to the effects of either MTL or PFC lesions (see [28]).

It remains unclear, however, what particular role, if any, these structures play in the input processes of encoding and consolidation and the output processes of retrieval for episodic and factual information. Many workers now believe that the MTL consolidates and stores (at least for a while) an index that links together the components of episodes (and possibly facts) that are represented in different neocortical sites (for example, see [29]). One possibility is that the MTL consolidates and stores at least temporarily an index that represents in memory certain kinds of associations [6, 15]. Episodes, and possibly facts, comprise information that includes these associations. There is not only extensive neuroimaging evidence that the MTL is activated during encoding [9,31] and that this activation is greater when encoding successfully produces later memory [3,35], but also that MTL activations are greater when inter-item associations rather than items are encoded [10,11,16–18].

ISSN 0953-4180/01/02/$8.00 © 2001/2002 – IOS Press. All rights reserved

This evidence is consistent with the above view that the MTL is critical for consolidating certain kinds of association into long-term memory. It seems probable that, if the MTL stores associative information, then, for as long as it does so, it should also mediate the retrieval of these associations and be activated when they are retrieved. If the MTL associative storage view is correct, then it would be expected that retrieval of recently learned associations should activate the MTL more than retrieval of well-established semantic associations, which all evidence indicates does not depend on the MTL.

Although MTL retrieval activations have been reported (see [4]), they have not been found consistently across studies. This inconsistency might have arisen for any or all of several reasons. First, the MTL may always be relatively active so that activity increases will be hard to detect. Second, retrieval may only activate a small proportion of MTL neurons so that the resultant cerebral blood flow changes (CBF) are hard to detect. Third, if baseline conditions contain novel items, MTL encoding activations may cancel the retrieval activations. Fourth, in accordance with the view that the MTL mediates retrieval of associations, retrieval of some kinds of information may involve minimal associative retrieval whereas retrieval of other kinds may involve high levels of associative retrieval. Finally, in studies that use block designs, significant activation may not be found, even when part of a memory is stored in the MTL and successful retrieval requires its reactivation, if the retrieval success rate is not sufficiently high. If this is correct, then, in block design studies, episodic retrieval should be associated with greater MTL activation when a higher proportion of items are successfully retrieved.

While it is well-established that the PFC is involved in the retrieval of episodic and semantic memories (see [13,15]), it is less clear to what extent and how the left and right PFC, and, relatedly, the specific PFC regions within each hemisphere, are functionally differentiated with respect to their roles in retrieval. With respect to possible differences between the roles in retrieval of the left and right PFC, several views have been advanced. The earliest view related to the neuroimaging literature emerged from the HERA model, which proposed that the left PFC is activated in both episodic encoding and semantic retrieval whereas the retrieval of verbal as well as non-verbal episodic memories principally activates the right PFC (see [33]). However, while this may be so, the left PFC has frequently also been activated (see [5] for a review) and, therefore, its potential role in episodic retrieval is of interest. According to HERA, these left PFC activations reflect semantic information retrieval. It has been suggested, however, that as episodic retrieval becomes more effortful and complex the activation of the left PFC increases relative to the right PFC [19]. For example, recall and recognition based on recollection should produce relatively more left PFC activation than recognition based on familiarity because it tends to involve more effortful and complex retrieval processes.

Episodic memory retrieval has been shown to activate many PFC regions, including the dorsolateral, ventrolateral, and anterior PFC regions (see [7]). These regions are also activated when information is encoded into episodic memory and during the performance of working memory tasks. Drawing on the available literature about the variables that influence these effects, Fletcher and Henson [7] have argued that the ventrolateral PFC is concerned with the updating and maintenance of information, the dorsolateral PFC with the selection, manipulation and monitoring of that information, and the anterior PFC with the selection of processes and subgoals relevant to these tasks. According to their view, the contribution of different PFC regions to retrieval depends upon the executive processes necessary for retrieval in a variety of circumstances. PFC laterality effects related to retrieval will relate to either the kind of information involved (verbal or hard-to-verbalize), the lateralization of some executive processes, or both. For example, it has been suggested that anterior PFC episodic retrieval activations are primarily right-sided, are found whether verbal or non-verbal information is involved, and may relate to specific kinds of expectancy (see [4]).

Even if PFC functional fractionation relates solely to different executive processes, it still needs to be resolved how these executive processes relate to retrieval variables that seem to influence the extent and location of PFC activations. Thus, it has been argued that there is a retrieval mode or mental set, which prepares the system for efficient retrieval (see [20,25]). This set presumably involves the executive control of attention that is likely to be at least partially mediated by the PFC. One issue is whether the retrieval set for episodic memory and well-established semantic memory is different. Retrieval set is also likely to be a relatively tonic process and should be detectable using block functional neuroimaging designs. In such designs, set can be kept constant, but retrieval effort varied across blocks. Retrieval effort is likely to increase when memory search needs to be carefully organized. This should be necessary when learning is poorer and memory is generally worse. However, level of effort is likely to be a phasic property of retrieval, which will vary to some extent from item to item. When the frequency of target items has been varied across blocks, it has sometimes been reported that certain PFC activations are greater when retrieval is more successful because of a higher target frequency (see [4,24]). Such effects could arise for three reasons, any or all of which may apply. First, they could arise for the same reason that might apply to the MTL, i.e. part of the memory is stored in a specific PFC region so this region is most activated when the memory is successfully retrieved. To our knowledge, this has never been claimed although the possibility should not be excluded. Second, retrieval success may lead to post-retrieval monitoring to check that the memory is accurate and appropriate to the situation [25]. Third, retrieval success is associated with a feeling of aware remembering (familiarity or recollection) and the PFC regions, the activation of which correlates with success, could be involved in producing this feeling. Buckner and Wheeler [4] have argued that a network of structures that includes the anterior PFC and several parietal cortex regions may provide the signal of "oldness", which produces a feeling of memory.

The aims of the present study were two fold. The first aim was to investigate the contribution made by the MTL during retrieval, and particularly how this contribution changes as a function of successful retrieval. The second aim was to clarify the role of the PFC in episodic retrieval by manipulating retrieval success as a function of memory strength rather than target density at test. When retrieval success is manipulated by varying memory strength, then lower success rates are likely to be associated with greater search effort.

## 2. Methods

### 2.1. Subjects

Twelve healthy right-handed subjects (age range 40 to 60) took part in this study, having given informed consent. The study had approval from the hospital ethics committee and ARSAC, Department of Health, UK.

### 2.2. Cognitive activation tasks

In order to isolate the episodic component of memory function, two separate tasks were developed that were different to, but based on, a design by Shallice et al. [27]. The experiment used an auditory cued recall design to contrast the processes of cued episodic recall and cued semantic recall. The baseline task, which involved retrieval from semantic memory, required subjects to generate words that were semantically associated with high frequency noun stimuli presented to them at a rate of one every five seconds. The demands of this task therefore involved retrieval of semantic associates only. The episodic memory task required subjects to recall the second word of a previously studied word pair in response to the presentation of the first word. The word pairs selected were weak semantic associates produced by 5%–8% of a sample population [23] and consisted of words of high frequency [14] and high concreteness (e.g., stream – fish, cat – fur, hand – ring). The presentation rate was matched to that of the baseline task. The demands of the episodic memory task therefore involved retrieval of both semantic and episodic information. This design ensured that while both tasks were similar in the cognitive demands they made with respect to verbal output and semantic processing, the episodic memory task would have involved additional retrieval of contextual information linked to the studied word pairs.

### 2.3. Memory training procedure

Specific memory training procedures were designed to manipulate processes relating to retrieval attempt (e.g. recollective effort) and retrieval success. Subjects were selected for one of two alternative training procedures. In the high performance procedure, subjects ($N = 6$) received repeated presentations (5 or 6) of the word pairs and a short presentation-test delay (approximately 10 minutes) producing high retrieval success. In the low performance procedure, subjects ($N = 6$) received limited presentations (1 or 2) of the word pairs and an extended presentation-test delay (approximately 30 minutes). This procedure produced a contrasting condition of low retrieval success and high recollective effort.

### 2.4. Neuroimaging procedure

Prior to each SPECT scan a maximum dose of 500MBq Tc99mHMPAO was injected intravenously through a forearm cannula while the subject performed the cognitive task. The tasks were designed to last for five minutes and the injection was administered 1.5 minutes after the beginning of each task. SPECT

Table 1
The first two contrasts show brain activations produced by the retrieval of episodic information compared to semantic information for the low performance group (LP) and the high performance group (HP) separately. The second two contrasts show which of these activations is greater for LP and then for HP

| Contrast | Region | B A | X | Y | Z | Size | Z-Score |
|---|---|---|---|---|---|---|---|
| LP Episodic – LP Semantic ($N = 6$) | Left middle frontal gyrus | 46/9 | −44 | 32 | 28 | 167 | 3.91 |
| | Left orbito-frontal | 11/47 | −14 | 38 | −24 | 121 | 3.04 |
| | Right middle frontal gyrus | 46/10 | 40 | 40 | 8 | 151 | 3.58 |
| HP Episodic – HP Semantic ($N = 6$) | Left inferior MTL | 36 | −16 | 0 | −32 | 151 | 3.01 |
| LP Episodic – HP Episodic ($N = 6 - N = 6$) | Left middle frontal/orbito-frontal gyrus | 46/9/45/11 | −42 | 32 | 28 | 541 | 4.27 |
| | Right middle frontal gyrus | 46/45 | 40 | 40 | 8 | 188 | 4.19 |
| HP Episodic – LP Episodic ($N = 6 - N = 6$) | Left inferior MTL | 28/36 | −32 | 0 | −28 | 104 | 3.66 |

imaging started approximately 5 minutes after injection, with the two SPECT scans being carried out 48 hours apart. The SPECT scans were acquired using an SME 810 multi-detector scanner [2].

### 2.5. Data analysis

SPM96 analysis [8] was used to explore task related differences. The co-registered SPECT images were converted to ANALYZE$^{\mathrm{TM}}$ compatible format. All SPECT scans were spatially normalised to the standard PET template provided with the SPM96 software and then smoothed with a 12 mm FWHM Gaussian filter. The spatially normalised and smoothed SPECT CBF images were adjusted for individual differences in global blood flow using proportional scaling to allow a direct comparison with an ROI analysis (results not presented here). Using the SPM96 software the significance of CBF differences between the two tasks was tested by calculating F-ratios, which were then converted to standard Z-scores. Only clusters of contiguous voxels equal to, or greater than 70, activated at the $p <$ 0.01 (uncorrected) level, with peak z-scores $>$ 3.0 and located within hypothesized regions were considered significant. The locations of local maxima were then expressed as co-ordinates in Talairach and Tournoux stereotaxic space [32] and Brodman areas (BA).

## 3. Results

### 3.1. Behavioural data

The episodic memory task behavioural data confirm that the manipulations of number of presentations and presentation-test delay produced two distinct levels of cued recall performance. The high performance group displayed a mean correct recall performance level of 95%, while the low performance group displayed a mean correct recall performance level of 55%. Performance on the semantic memory task was very high and matched across groups, with an overall mean of 99.6%.

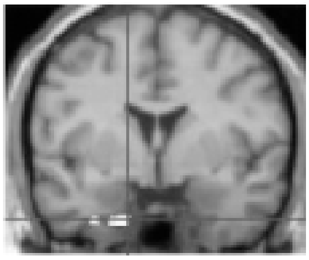

Fig. 1. Left inferior medial temporal lobe activation produced by the highly successful retrieval of episodic information compared to the retrieval of semantic information.

### 3.2. CBF data

The experiment produced CBF data for both semantic and episodic retrieval conditions but only the episodic retrieval contrasts will be reported here. Using the subtraction paradigm, CBF results were analysed for the following contrasts. The first CBF contrast involved a within group comparison of the activation pattern produced by episodic retrieval compared to semantic retrieval, for the high and low performance groups separately, This contrast produced a left inferior medial temporal lobe activation (BA36, $Z = 3.01$) in the high performance group and a bilateral dorsolateral frontal activation (BA 46/9/10, $Z = 3.91$ and 3.58) and a left-sided orbito-frontal activation (BA 11/47, $Z =$ 3.04) in the low performance group (see Table 1 and Figs 1 and 2.)

The second CBF contrast involved a between group comparison of the episodic-semantic activation patterns. This analysis involved the subtraction of the high performance (episodic-semantic) from the low performance (episodic-semantic) to produce a resultant low performance episodic activation pattern and the subtraction of the low performance (episodic-semantic) from the high performance (episodic-semantic) to produce a resultant high performance episodic activation pattern. The low performance episodic activations were again in the bilateral frontal regions; on the left extend-

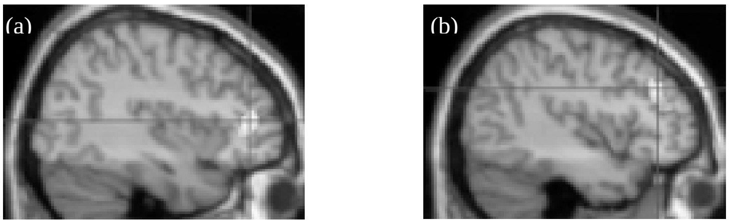

Fig. 2. Right (a) and left (b) frontal lobe activations produced by poor episodic retrieval compared to semantic retrieval.

ing into BA 46/9/45/10 ($Z = 4.27$) and on the right into BA 46/9/45 ($Z = 4.19$). This time, however, the right-sided activation did not extend the into BA10 anterior region and the left orbito-frontal activation was not reported separately, but as part of a very large ($>$ 500 voxel) left-sided frontal activation. The high performance episodic activation pattern revealed a similar inferior medial temporal lobe activation (BA 28/36, $Z = 3.66$) to that reported above (see Table 1 and Figs 3 and 4.)

It is worth noting that the high performance contrasts that produced the medial temporal lobe activations, produced no additional activations at the uncorrected threshold of $p = 0.01$. However, the low performance contrasts that produced the bilateral frontal activations also revealed two much smaller activations in the right anterior cingulate cortex (BA 24) and the right medial frontal cortex (BA9).

## 4. Discussion

The overall results of the study are clear. Successful cued recall, found when good learning had led to strong memory, produced significant left MTL activation whereas less successful cued recall, found when minimal learning had led to weak memory, failed to produce significant MTL activation. Furthermore, left MTL activation in the same region was greater when cued recall was more successful rather than less successful. In contrast, effortful cued recall, that was probably present when minimal learning led to weak memory and poor recall, produced significant bilateral PFC activations in regions that primarily included the dorsolateral PFC, but which extended into the ventrolateral PFC and even the anterior PFC. However, the less effortful cued recall, which was probably associated with strong memory and better performance, failed to activate any PFC region significantly even at a reduced threshold. Furthermore, more effortful and less successful cued recall produced significantly more activation in a broadly similar set of PFC regions than did less effortful, more successful cued recall.

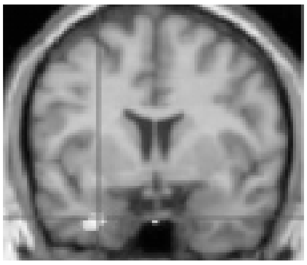

Fig. 3. Left inferior medial temporal lobe activation produced by the highly successful retrieval of episodic information compared to the less successful retrieval of episodic information.

Our MTL findings are similar to those of Schacter et al. [26], who reported a right MTL activation in subjects showing good cued recall following effective learning. The similarity indicates that the relationship between MTL activation and success frequency in block design emission tomography studies has some generality because Schacter et al.'s study differed from ours in a number of important ways. They used word stem cued recall, manipulated performance by contrasting semantic with non-semantic orienting tasks, and their criteria for high and low performance were different from those used here. Both our study and that of Schacter and his colleagues found a different pattern of MTL results from a study by Petersson et al. [21,22]. In this study, a similar but bilateral, MTL activation was produced when less-well trained subjects were compared with well-trained subjects on an abstract drawing recall task.

The results of Petersson et al.'s study and the other two studies may differ for one or more of several possible reasons. First, retrieval is associated with encoding as is indicated by the ability of people to remember what they did during earlier retrieval attempts even when these were unsuccessful. Encoding that leads to better later memory activates the MTL more [3,35]. So, if memory for what was done during unsuccessful retrievals was better than memory for what was done

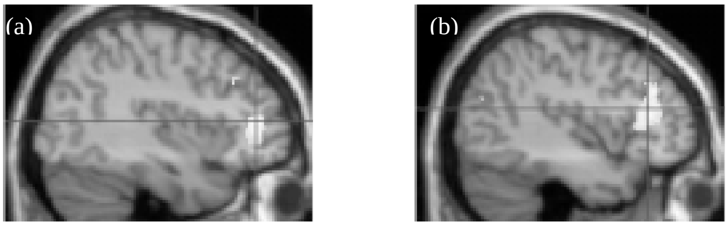

Fig. 4. Right (a) and left (b) frontal lobe activations produced by poor episodic retrieval compared to highly successful episodic retrieval.

during successful retrievals in Petersson et al.'s study, but not the other two, then unsuccessful retrieval might produce greater MTL activations. A related possibility is that unsuccessful retrieval involves more processing and so more can be remembered about each retrieval attempt. This would also lead to more MTL activation. Checking these possibilities will not be easy in the context of a functional neuroimaging study.

Second, different MTL regions may display different functional relationships between memory strength and activation when retrieval is successful. This could explain the different pattern of results in the three studies if the study of Petersson et al. involved activations in different MTL regions from the other two studies. Our study lacks the spatial resolution to identify whether the MTL activation involves the hippocampus, the MTL cortices (particularly the perirhinal and entorhinal cortices), or both. Lesion evidence (see [15]) suggests that all these regions should be activated when verbal associations are recalled. So examination of this second possibility will require the use of an imaging procedure with sufficient spatial resolution to determine whether the hippocampus and MTL cortices show different memory strength-activation relationships.

The third possibility is not incompatible with the other two and is perhaps the most interesting. Neither our study nor that of Schacter and his colleagues indicates that MTL activation increases as memory becomes stronger and/or retrieval becomes more fluent and effortless. Rather, the studies indicate that MTL activation increases as the proportion of successful retrievals in a scanning block also increases. In contrast, the study of Petersson and his colleagues strongly suggests that MTL activation decreases as memory becomes stronger and/or retrieval becomes more fluent and effortless. The suggestion is particularly strong because the effect of memory strength and/or fluency has to be significantly stronger than an effect in the opposite direction related to the proportion of successfully retrieved items. It is possible that, at lower levels of memory strength and/or fluency of retrieval, MTL activation levels do not change as strength and/or fluency increase, but that, at higher levels, MTL activation begins to decrease as strength and/or fluency increases. This possibility is weakly compatible with the somewhat higher levels of retrieval found in the Petersson et al. study relative to the other two studies. Exploration of whether a non-linear strength/fluency-MTL activation function of this kind exists will require the use of a parametric event-related fMRI procedure in which memory strength/retrieval fluency is systematically varied through the use of differing numbers of training trials and/or amount of retrieval practice. Such a study would only examine activations produced by successful retrievals of memories that systematically vary in strength and/or fluency. This procedure should avoid confounding variations in memory strength (i.e. how strong and/or rich memories of successfully retrieved individual items are) with variations in retrieval success (i.e. what proportion of individual items are successfully retrieved where richness and strength of retrieved memories is not a factor). Possible changes in what is retrieved as memory gets better would also need to be controlled. If a non-linear function is found, then it will become important to determine to what extent it is specific to certain MTL regions, whether it is a function of differential encoding effects that lead to retrieval of memories differing in strength or fluency of retrieval, or whether it has another explanation.

Our PFC results are similar to those of Schacter et al. [26], Petersson et al. [21,22] and also Andreasen et al. [1], who found greater PFC activations bilaterally following unpracticed (and presumably more effortful) recall of a word list relative to practiced word list recall. Our preferred interpretation of the greater bilateral PFC activations found in the subjects with weaker memory and less successful retrieval is that these regions mediate executive processes that are involved in retrieval search and that these regions are more active because more effort has to be exerted when memory is weaker (see also [4]). The greater PFC activations were found to lie more extensively in the left hemisphere and particularly in dorsolateral PFC. The dorsolateral PFC focus is consistent with Fletcher and Henson's [7] view that this region mediates the executive processes of selection and manipulation of information. The left sided bias is consistent with evidence that more effortful retrieval produces relatively greater left PFC activations [19]. Further and more direct support for our interpretation is needed. Event-related fMRI could be used to separate successful and unsuccessful retrievals from each other. Such separation is important because some PFC retrieval activations are claimed to be success related (see [4]). The event-related procedure would also allow more direct measures of effort, such as changes in heart rate or pupil dilation or even reaction time, to be used so that effort levels could be categorized on the basis of these measures.

Another possible explanation is that the subjects with stronger memory are often able to retrieve the target response words immediately when given the stimulus word cue whereas the subjects with weaker memory would more often have recalled several semantic associates whether or not they were eventually successful at retrieving the episodic target. On this view, subjects with poor memory would have retrieved far more semantic memories than the high performance subgroup causing more activation of the left PFC as this region is believed to be activated when semantic memories are retrieved [34]. Several comments on this possible explanation are warranted. First, retrieval of semantic memories is not a necessary condition for activation of the left PFC region. Thus, while left PFC activations have been reported in similar conditions of low performance [21,22,26] these studies have not required as much semantic retrieval as the current study, and, therefore, their activations are unlikely to reflect semantic retrieval. Second, there is evidence that retrieval of more episodic as well as more semantic information is associated with greater activation of the PFC [12]. This evidence indicates that greater right as well as left PFC activation (although in non-mirror image sites) is associated with the retrieval of more information regardless of whether this information is semantic or episodic. It is, therefore, possible that, in our study, retrieval of less well trained memories was associated with greater right and left PFC activations because subjects were recalling more semantic associates, more episodic memories (such as the context of the study situation), or both. Finally, as the subjects' recall was intentional, the retrieval of more episodic and/or semantic information will almost certainly be associated with greater levels of effortful search processing. The second possible explanation is, therefore, most probably a specific variant of the our preferred explanation.

Our study compared the activating effects of the cued recall of paired response words linked to episodes with that of the cued recall of response words linked in well-established semantic memory. The bilateral PFC activations that were greater for the episodic cued recall in the weak memory subjects are very unlikely to be related to the kind of information being retrieved. In other words, the PFC effect probably is not a direct function of the retrieval of episodic versus semantic information. It is more likely to relate to the amount of effort involved in retrieval regardless of the kind of information that is being retrieved. Cued recall of overlearned and well-established semantic information probably involved very little effort. Similarly, subjects with strong memory were able to retrieve episodic memory with little effort and these subjects showed no PFC activation differences when their episodic and semantic retrieval was compared. Therefore, although retrieval of semantic and episodic memories may sometimes be associated with different PFC activations, our study finds no clear evidence for such effects.

Interestingly, although an anterior area of the PFC on the right was more activated by episodic than semantic cued recall in the subjects with weak memory and not in the subjects with strong memory, there was not a significant difference in the size of this effect between the two groups. Provided the effect would not have become significant with a larger group of subjects, this is consistent with Buckner and Wheeler's [4] conclusion that right anterior PFC retrieval activations produced by episodic retrieval are not modulated by either success or effort.

In our study, no PFC region was more activated in the group of subjects with good memory than in the group with poor memory and less successful recall. Many studies have, however, found success related PFC retrieval activations and it is possible that our study would have found success-modulated PFC retrieval activations if we had included more subjects. However, an event-related fMRI procedure would be more appropriate because it would allow comparison of successful and unsuccessful retrievals for memories that systematically differ in strength.

In conclusion, therefore, our study suggests that the roles of the MTL and PFC in episodic verbal retrieval are distinct. Although more evidence is needed, our results are consistent with the view that the MTL becomes active in retrieval because it stores aspects of the episode that are successfully retrieved. In contrast, the dorsolateral region of the PFC bilaterally, and possibly other PFC regions as well, seem to be involved with executive processes that mediate the search processes that underlie recall whether or not it is successful. This conclusion is compatible with both the MTL and the PFC subserving several functions relevant to memory.

## Acknowledgements

We would like to thank Mary-Theresa Hansen and Marion Cresswell for their assistance with SPECT scanning. This study was funded by The Wellcome Trust.
